# Projecting suitable habitats and prioritizing conservation areas for *Dendrobium shixingense* under climate change

**DOI:** 10.3389/fpls.2025.1620580

**Published:** 2025-08-08

**Authors:** Wei Lin, Yingying Ren, Guochun Fan, Min Deng, Yibing Liu, Qingxia Zhang, Xiangxin Xu, Shiting Huang, Hua Zhang, Junsheng Qi

**Affiliations:** ^1^ College of Biology and Food Engineering, Chongqing Three Gorges University, Chongqing, China; ^2^ College of Environmental and Chemical Engineering, Chongqing Three Gorges University, Chongqing, China; ^3^ Green Planting and Deep Processing of Genuine Medicinal Materials in Three Gorges Reservoir Area, Chongqing Three Gorges University, Chongqing, China

**Keywords:** *Dendrobium shixingense* Z. L. Chen, S. J. Zeng & J. Duan, Suitable area, MaxEnt, Marxan, InVEST, priority protected area

## Abstract

*Dendrobium shixingense* Z. L. Chen, S. J. Zeng & J. Duan, a National Class II Protected wild plant species in China, is renowned for its rich polysaccharide content and remarkable medicinal value. Delineating priority conservation areas for this species is critically important for its sustainable conservation and management. In this study, the MaxEnt model was applied to predict its potential distribution patterns under multiple climate scenarios, while the Marxan and InVEST models were utilized to identify priority conservation zones. Results demonstrate that the primary distribution of *D. shixingense* is concentrated in southeastern China, particularly within Guangdong, Fujian, Guangxi, and Jiangxi provinces, with a total suitable habitat area of 79.41 × 10^4^km^2^. Future projections indicate an expansion of suitable habitats, with key environmental drivers identified as precipitation of the coldest quarter (Bio19), mean diurnal temperature range (Bio2), among others. Priority conservation areas are predominantly located in Shixing County and Ruyuan Yao Autonomous County of Shaoguan City; Xing’an County of Guilin City and other specified regions. These findings indicate that climate change will substantially impact the distribution of *D. shixingense*, potentially altering both the extent and quality of suitable habitats. priority conservation areas are concentrated in ecologically stable regions, necessitating enhanced protection efforts in these zones. Collectively, this research provides a robust scientific foundation for formulating effective conservation strategies and advancing the sustainable development of *D. shixingense*.

## 1 Introduction

Global biodiversity continues to decline at an unprecedented rate (Pereira et al., 2012), with climate change emerging as one of the most formidable challenges for conservation in the 21st century (Ceccarelli and Grando, 2020). Species distributions themselves arise from intricate interactions among evolutionary processes, anthropogenic activities, and environmental drivers (including climatic, topographic, edaphic, and biotic factors), collectively reflecting population dynamics and ecological adaptations (Soberon and Peterson, 2005). Understanding the dynamic interplay between species distribution shifts and climate change is therefore pivotal for biodiversity conservation and sustainable ecosystem management (Li et al., 2024). This urgency is underscored by the observed increase in global mean surface temperature (1.1°C for 2011–2020 relative to 1850-1900) and projected future warming, which exhibits significant divergence across emission scenarios—stabilizing at 1.5°C under stringent mitigation but potentially reaching 4.4°C if high emissions continue (Pirani et al., 2024). Critically, a 1.5°C warming threshold alone may place 3-14% of terrestrial species at critically high extinction risk (Ma et al., 2022). These climate-driven changes profoundly impact community composition and alter species distribution patterns (Bosso et al., 2017), though shifts vary across taxonomic groups.

Species distribution models (SDMs) quantify ecological niches through regression and classification algorithms that correlate occurrence records with environmental variables (Phillips et al., 2006). By projecting these relationships onto climate scenarios, SDMs estimate habitat suitability through probabilistic surfaces. Widely applied in ecology, invasion biology, and conservation science, prevalent SDM approaches include Maximum Entropy (MaxEnt), Genetic Algorithm for Rule-set Production (GARP), Ecological-Niche Factor Analysis (ENFA), and BIOCLIM models (Townsend Peterson et al., 2007; He et al., 2023). While GARP tends to overestimate species distribution ranges (exhibiting high false positive rates) (Townsend Peterson et al., 2007), ENFA is excessively sensitive to extreme environmental values at niche margins (Hirzel et al., 2006), and BIOCLIM fails to account for variable interactions (Beaumont et al., 2005), the MaxEnt framework demonstrates particular efficacy under data-limited conditions (n≥5) – a critical advantage for modeling narrow-range species – achieving robust predictions through entropy maximization principles (Phillips and Dudík, 2008). To translate these suitability predictions into actionable conservation plans for spatially restricted species, we integrate MaxEnt outputs with systematic reserve design tools (Chen et al., 2025). The Marxan model employs simulated annealing algorithms to design cost-efficient reserve networks meeting user-defined conservation targets (Smith et al., 2010), extending from its marine origins to terrestrial conservation planning (Zhang and Li, 2022). However, while Marxan optimally identifies minimal conservation units, it neglects habitat quality assessment – a critical limitation given that habitat quality directly determines species persistence, demographic rates, and long-term viability (Newbold et al., 2015). Degraded habitats constrain dispersal corridors and reduce resilience to climate stressors, particularly for range-restricted species (Damschen et al., 2019). This gap is addressed by the InVEST model’s habitat quality module, which enables spatially explicit ecosystem evaluation with minimal data requirements (Kunwar et al., 2020). This study integrates Marxan’s systematic planning with InVEST’s habitat quality assessment to refine priority areas, particularly when combined with MaxEnt-derived medicinal plant suitability predictions.

*Dendrobium shixingense* Z. L. Chen, S. J. Zeng & J. Duan, a newly described orchid species published in 2010 (Chen et al., 2010), is a nationally protected wild plant (Category II under China’s Key Protected Wild Plants List) and listed in CITES Appendix II. Endemic to Shixing County, Shaoguan City, Guangdong Province, this epiphytic orchid derives its name from its type locality. Within the genus Dendrobium Sw., which encompasses numerous species with complex taxonomic identities and medicinal applications, *D. shixingense* is highly valued in traditional medicine for its rich polysaccharide content and purported therapeutic properties, including yin-nourishing, qi-tonifying, and anti-aging effects (Wang et al., 2025). Although advances in tissue culture techniques, seed germination protocols, and rapid propagation systems have enabled successful establishment of its regeneration framework (Meng et al., 2012; Wang et al., 2025), the species faces severe conservation challenges. Escalating abiotic stressors (e.g., extreme heat and drought) driven by climate change increasingly threaten its natural habitats, while unsustainable harvesting has pushed wild populations to the brink of collapse. This study aims to: (1) Predict climate-driven shifts in *D. shixingense’s* potential suitable habitats using MaxEnt modeling; (2) Identify key environmental determinants shaping its distribution patterns; (3) Delineate priority conservation areas by integrating Marxan’s spatial optimization with InVEST-based habitat quality assessments. The findings will provide scientific support for developing targeted conservation strategies and sustainable management of this critically endangered medicinal resource.

## 2 Materials and methods

### 2.1 Species occurrence data

**Figure 1:**
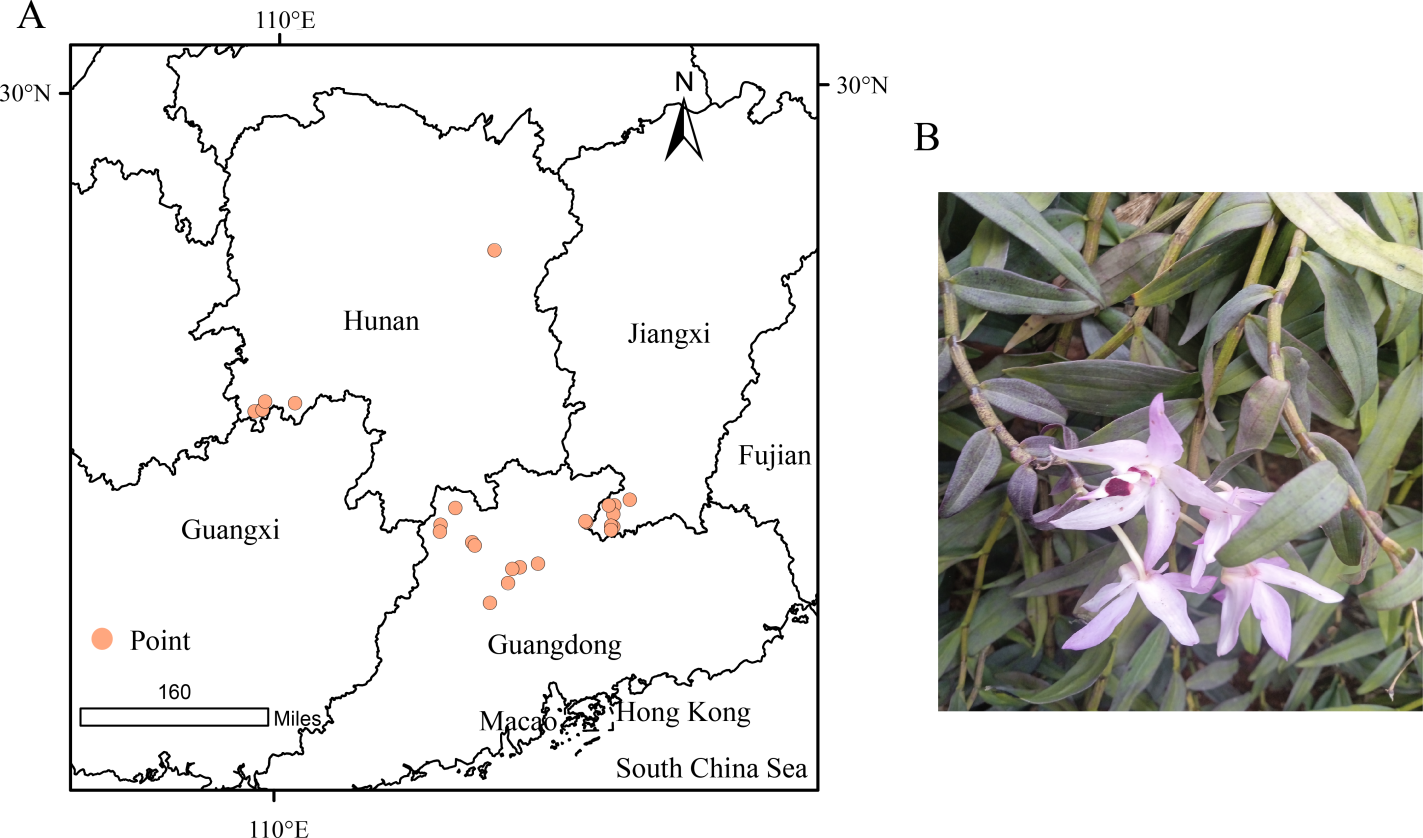
Geographical distribution point of *D.shixingense* **(A)**, Illustration of *D.shixingense* **(B)**.

*D.shixingense* displays pink flowers with a labellum featuring purple-tinged calli, a column bearing pale violet hues, and a purplish anther cap, demonstrating morphological comparability to *Dendrobium officinale*. To obtain accurate distribution records of *D.shixingense*, we first compiled occurrence data from the Chinese Plant Science Center and published literature, followed by field surveys. A total of 53 georeferenced occurrence points were initially collected. To mitigate potential biases caused by spatial autocorrelation, we filtered the dataset using the ENMtools package in R, retaining 26 spatially independent points for subsequent analyses (**Figure 1**).

### 2.2 Environmental variables

Climatic Data: Nineteen bioclimatic variables (Bio1–Bio19) were obtained from the WorldClim database (https://worldclim.org) at 30-arcsecond (~1 km) resolution, covering historical (1970–2000) and future periods (2021–2040, 2041–2060, 2061–2080). Future climate projections were derived from the BCC-CSM2-MR model under three Shared Socioeconomic Pathways (SSPs): low-emission (SSP126), moderate-emission (SSP245), and high-emission (SSP585) scenarios, selected for their demonstrated reliability in simulating East Asian climate dynamics (Tan et al., 2022). Topographic and Edaphic Data: Altitude data (90 m spatial resolution) were acquired from the Geospatial Data Cloud (http://www.gscloud.cn/). Soil properties were extracted from the Harmonized World Soil Database (HWSD; https://gaez.fao.org/pages/hwsd). Land use data of China (2022) at 30-m resolution were sourced from the national-level Resource and Environmental Science Data Center (RESDC) (https://www.resdc.cn/).

**Figure 2:**
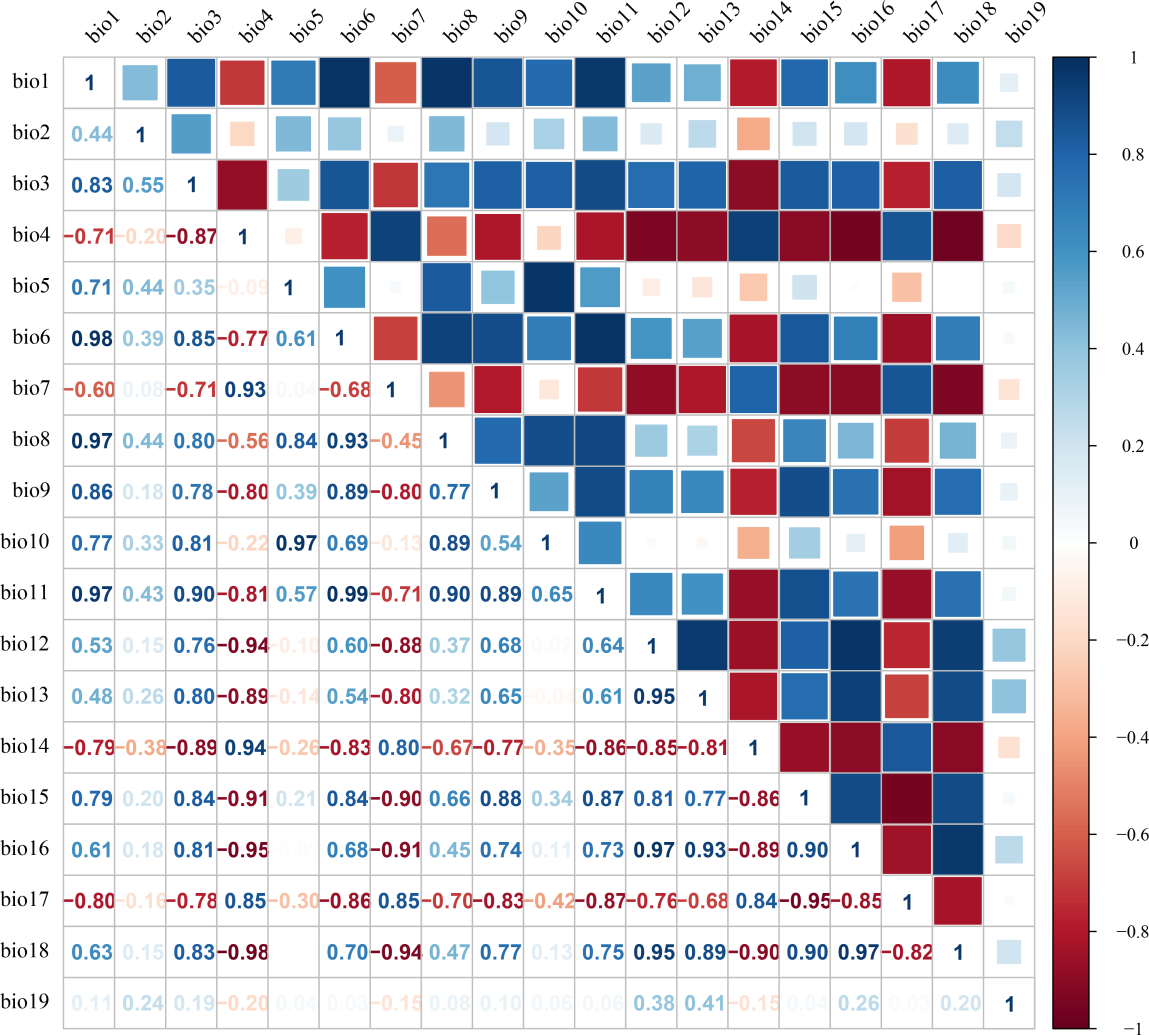
Spearman correlation analysis of bioclimatic factors.

**Figure 3:**
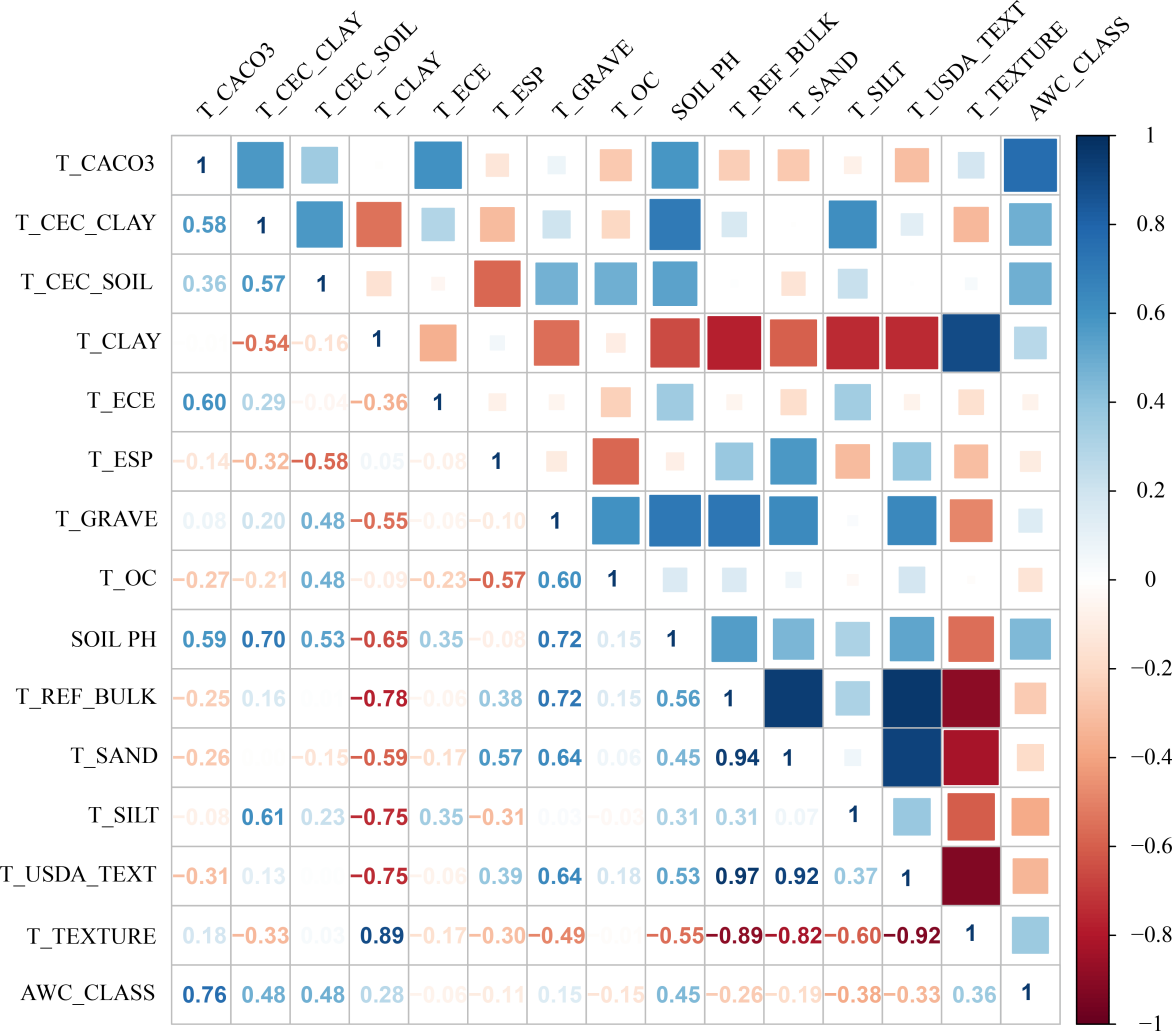
Spearman correlation analysis of soil factors.

Environmental variables may exhibit significant multicollinearity (Gong et al., 2022). To mitigate the impact of spatial correlation among predictors on MaxEnt model accuracy, we employed a sequential approach combining Jackknife tests and Spearman correlation analyses. First, bioclimatic and soil variables were separately imported into MaxEnt with the Jackknife test enabled to quantify their percentage contributions to model performance (Phillips et al., 2006). Subsequently, Spearman correlation analyses were conducted for both variable categories (**Figures 2**-**3**), with |R| ≥ 0.8 defined as indicative of high collinearity (Yang et al., 2023b; Jia et al., 2024). Finally, variables contributing <2% in Jackknife evaluations were discarded. For variable pairs showing high correlation (|R| ≥ 0.8), only the predictor with the greater explanatory contribution was retained (Song et al., 2023). This procedure yielded 4 bioclimatic, 5 edaphic and 2 topographic variables for final modeling (**Table 1**).

**Table 1:** Variables used in modeling.

Type	Variable	Description	Unit
Climatic	bio2	Mean Diurnal Range	°C
	bio3	Isothermality	–
	bio9	Mean Temperature of Driest Quarter	°C
	bio19	Precipitation of Coldest Quarter	mm
Topographic	Al	Altitude	m
	Sl	Slope	°
Soil	T_CEC_CLAY	Cation Exchange Capacity of the Clay Fraction	cmol_(+)_;/kg
	T_CLAY	Clay Content	%
	T_OC	Organic Carbon Content	%
	T_GRAVE	Gravel Content	%
	Soil pH	Soil pH	–

### 2.3 MaxEnt model parameter tuning

The use of default parameters in Maxent models may lead to overcomplexity, whereas the ENMeval package in R software effectively optimizes model parameters (Kass et al., 2021). We established regularization multiplier (RM) groups ranging from 0.1 to 4 with increments of 0.5, along with six feature combination (FC) parameters: H, L, LQ, LQH, LQHP, and LQHPT, where L (Linear), Q (Quadratic), H (Hinge), P (Product) and T (Threshold) represent distinct feature types (Tian et al., 2023). The Akaike Information Criterion corrected for small sample sizes (AICc) was utilized to evaluate model complexity and transferability. The optimal model was identified when the AICc increment minimized (delta.AICc = 0), while models with AICc < 2 were considered statistically credible (Phillips et al., 2017; Li et al., 2023).

Based on optimized parameters, model prediction employed the Bootstrap method for data partitioning. For each iteration, 25% of the data were randomly selected as the test set, with the remaining 75% used for model training. This process was replicated 10 times to mitigate stochastic errors caused by data partitioning, thereby enhancing the accuracy and stability of model evaluation (Sreekumar and Nameer, 2022). To systematically assess model performance, a Jackknife test was conducted to quantify the relative importance of environmental variables. Model accuracy was validated using the area under the receiver operating characteristic curve (AUC), where higher values (range: 0–1) indicate superior predictive performance, with AUC > 0.9 considered indicative of robust model simulations (Swets, 1988; Jiménez-Valverde, 2012). The Kappa statistic, which integrates species distribution rates, predictive sensitivity, and specificity, was adopted to evaluate model performance. Models are generally deemed reliable when Kappa values exceed 0.4 (West et al., 2016). However, this metric is susceptible to biases caused by species distribution frequency. In contrast, the True Skill Statistic (TSS), an improved evaluation index with a simpler computational framework, is widely recommended. A TSS value > 0.6 typically serves as the threshold for qualifying model performance (Zhao et al., 2021).

### 2.4 Suitability classification

The Maxent model prediction results were visualized using ArcGIS 10.8. The potential suitable habitats for *D. shixingense* were classified into four grades using the natural breaks method (Jenks) (Zhao et al., 2025): non-suitable (0–0.098), low suitable (0.098–00.329), moderately suitable (0329–0.612), and highly suitable (0.612–1). Additionally, the SDM toolbox extension in ArcGIS was employed to analyze dynamic changes and centroid shifts in suitable habitats under different climate change scenarios (Wang et al., 2023).

### 2.5 Marxan model

Biodiversity conservation prioritizes optimal resource allocation strategies that maximize threatened species coverage while ensuring population viability (Zhao et al., 2016). Priority conservation area delineation, essential for maintaining biodiversity and sustainability, requires systematic approaches (Margules and Pressey, 2000). The Marxan model, designed and developed by Ian Ball and Hugh Possingham (Ball and Possingham, 2000), was utilized to identify priority conservation areas for *D. shixingense* under current climatic conditions. The study area was divided into 2 km × 2 km planning units. Using the Zonal Statistics as Table tool in GIS, the distribution area of suitable habitats within each planning unit was quantified to construct a species distribution matrix (Kim and Choe, 2024). Approximately 20% of the total suitable area was set as the conservation target. The boundary length of planning units was generated using the ArcMarxan2.pyt plugin. Model parameters included 1,000,000 iterations (Yu et al., 2023), a boundary length modifier (BLM) of 10,000 to optimize spatial compactness, a species penalty factor (SPF) of 100 and 100 independent runs.

### 2.6 Habitat quality assessment using the InVEST model

The InVEST (Integrated Valuation of Ecosystem Services and Tradeoffs) model, developed by the Stanford Natural Capital Project and the World Wildlife Fund (Daily et al., 2009), was applied to assess habitat quality by integrating land-use types, threat sensitivity, external threat intensity, and spatial effects (distance and weight decay) (Deng et al., 2021). Building on Marxan-derived priority conservation areas, the InVEST model refined habitat quality evaluations. Threat factors, including paddy fields, drylands, urban areas, rural settlements, other construction lands, and unused lands, were selected based on the InVEST user manual (Sharp et al., 2020) and relevant studies (Shang et al., 2021; Feng et al., 2022). Threat factor weights, maximum influence distances and decay types were configured (**Tables 2** and **3**).

**Table 2:** Threat factor weight of study area.

Threat	Maximum impact distance (km)	Weight	Decay type
Paddyfield	4	0.7	linear
Dryland	3	0.5	linear
Urban	8	1	exponential
Village	5	0.6	exponential
other construction land	8	0.4	exponential
Unusedland	6	0.5	linear

**Table 3:** Sensitivity of land scape types to threat factors.

Land type	Habitat	Paddyfield	Dryland	Urban	Village	Other	Unusedland
Paddyfield	0.3	0	0.3	0.6	0.5	0.4	0.4
Dryland	0.3	1	0	0.6	0.5	0.5	0.4
Woodland	0.9	0.6	0.5	0.75	0.6	0.7	0.2
Shrub	1	0.6	0.6	0.8	0.7	0.7	0.2
Sparsewood	0.85	0.9	0.7	0.9	0.8	0.8	0.2
Other forest land	0.9	0.7	0.7	1	0.9	0.8	0.2
High coverage grassland	0.85	0.8	0.7	0.6	0.55	0.6	0.6
Medium coverage grassland	0.7	0.7	0.7	0.7	0.6	0.7	0.7
Low coverage grassland	0.7	0.6	0.7	0.8	0.7	0.8	0.8
Graff	1	0.8	0.65	0.85	0.7	0.5	0.3
Lake	0.9	0.3	0.3	0.7	0.6	0.6	0.3
Reservoir	0.7	0.7	0.7	0.85	0.7	0.5	0.3
Shoal	0.8	0.5	0.7	0.7	0.2	0.5	0.3
Urban	0	0	0.1	0	0	0	0
Village	0	0	0.1	0	0.5	0	0.1
Other	0	0	0	0.2	0.1	0	0
Swamp land	0.5	0.5	0.5	0.6	0.3	0.3	0.2
Unusedland	0	0	0	0	0	0	0

## 3 Results and analysis

### 3.1 Model accuracy and *D. shixingense* distribution

**Figure 4:**
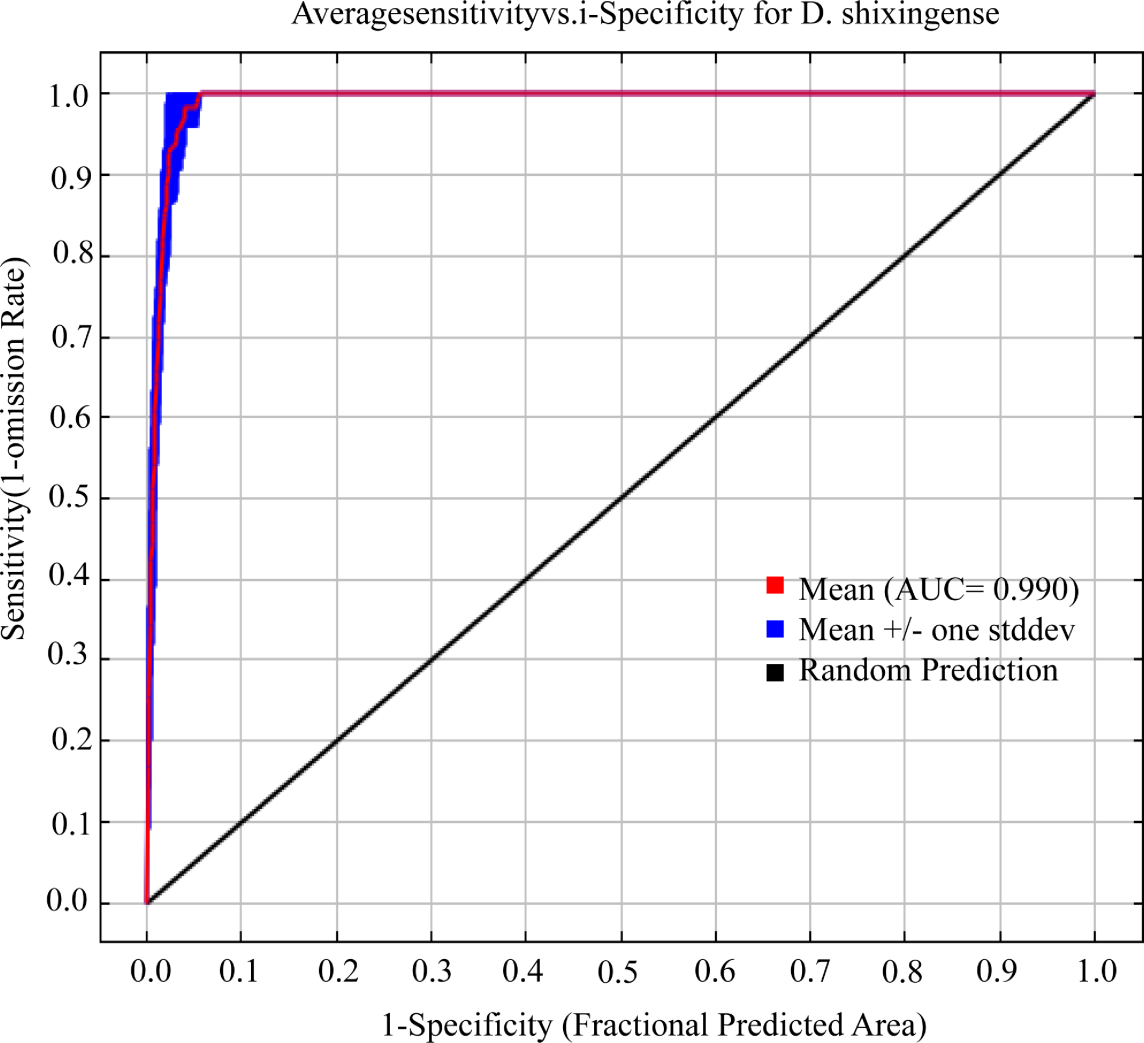
ROC curve for current climate prediction of *D. shixingense*.

Using default parameters in the Maxent model for predicting suitable habitats of *D. shixingense* yielded a delta.AICc value of 130.47. After parameter optimization via the ENMeval package, the optimal parameter configuration (feature combination [FC] = LQ, regularization multiplier [RM] = 1) achieved the minimal AICc increment (delta.AICc = 0). Under these settings, the model exhibited excellent predictive performance (**Figure 4**), with an AUC value of 0.990, expressing excellent (Swets, 1988), a TSS value of 0.932 expressing outstanding (Zhao et al., 2021), and a Kappa statistic of 0.477 expressing acceptable (West et al., 2016).

**Figure 5:**
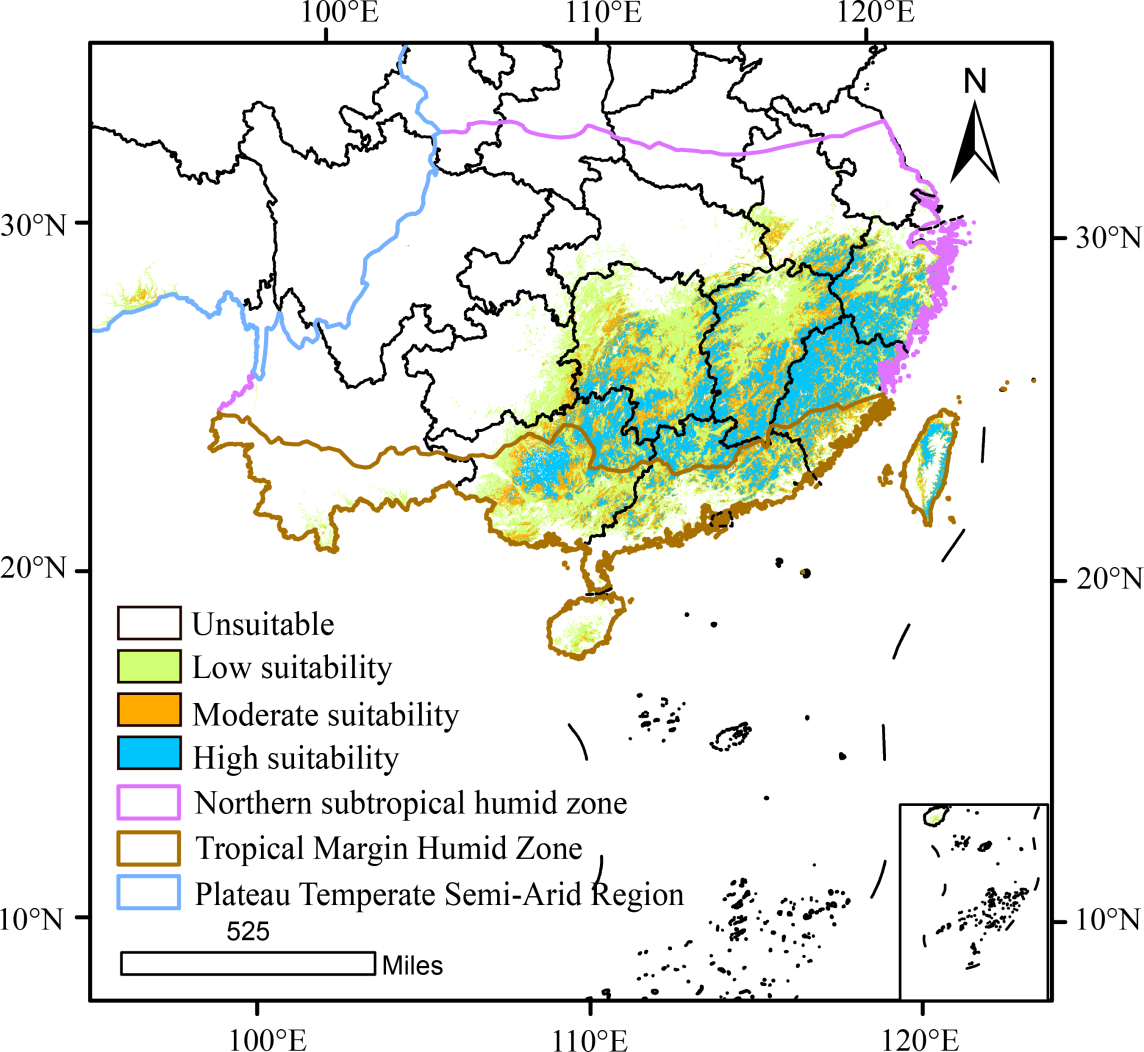
Suitable area for *D. shixingense* under current climate.

Visualization of the current climatically suitable habitats for *D. shixingense* in GIS revealed a concentrated distribution in southeastern China, covering a total area of 79.41×10^4^ km² (**Figure 5**). These habitats predominantly align with two climatic zones: the marginal tropical humid region and the northern subtropical humid region. Notably, moderately to highly suitable areas were primarily located in the northern subtropical humid zone. Specifically, the highly suitable areas (18.27×10^4^ km²) encompassed Guangdong, Fujian, Guangxi, and Jiangxi provinces. Moderately suitable areas (22.65×10^4^ km²) included Zhejiang and Guangxi provinces, while low-suitability zones (38.49×10^4^ km²) extended to Hunan Hainan and Jiangsu provinces in China.

### 3.2 Key environmental variables

This study investigated the interactions between the occurrence probability of *D. shixingense* and environmental factors, along with their response relationships, based on the contribution rates of these variables. Using the MaxEnt model, we prioritized the importance of environmental variables to define suitable habitats and identify key factors driving the species’ distribution. First, among the 11 variables analyzed, the most critical factors influencing habitat suitability were bio19 (precipitation of the coldest quarter, 32.5%), bio2 (mean diurnal temperature range, 19.9%), altitude (16.5%), Soil pH (14.6%), and bio9 (mean temperature of the driest quarter, 5.1%), with a cumulative contribution rate of 88.6%.

**Figure 6:**
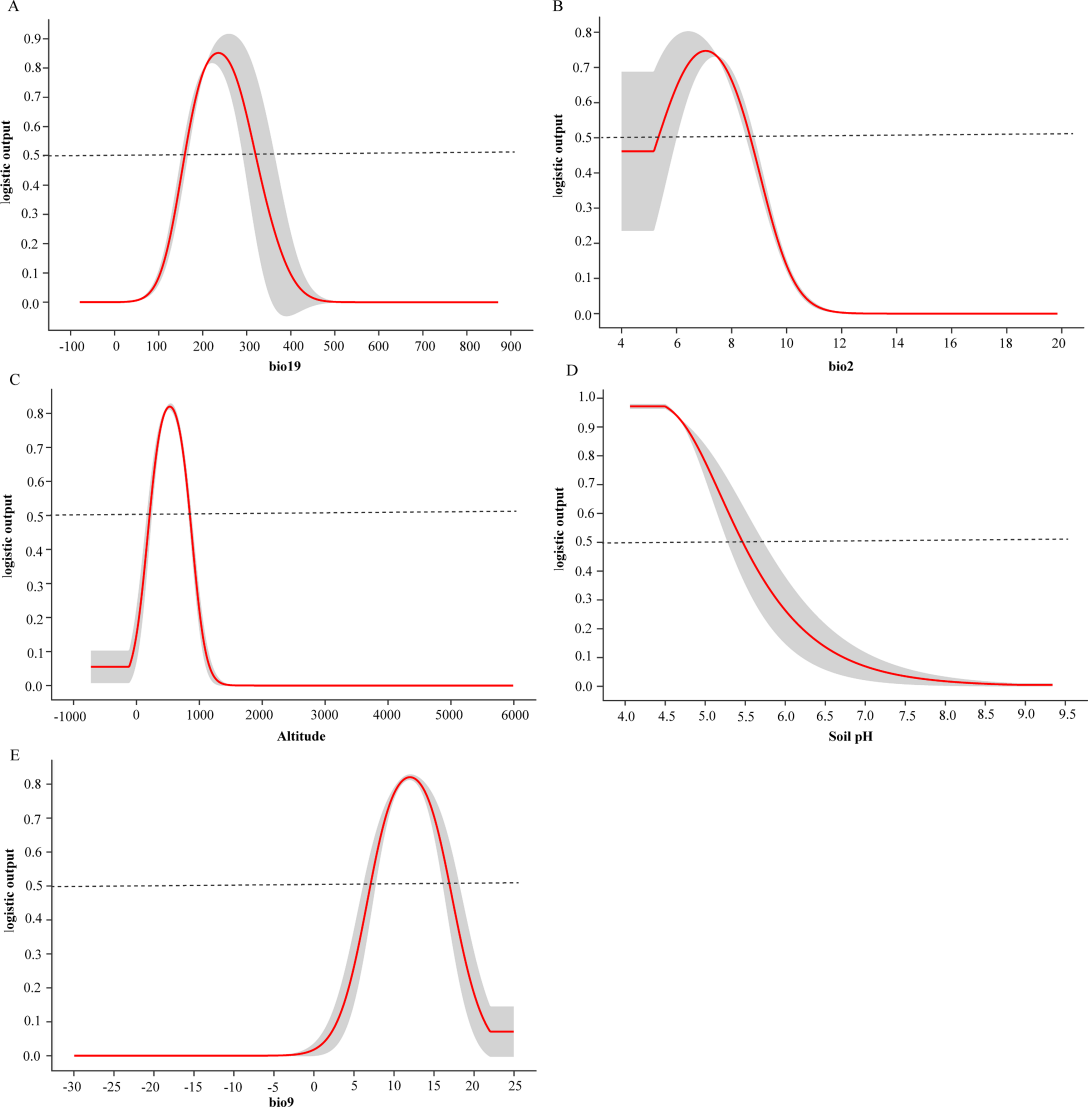
Single factor response curve of the current climate. **(A)** bio19, **(B)** bio12, **(C)** Altitude, **(D)** Soil pH, **(E)** bio9.

Secondly, for the five key variables, the model provides the response curve of each factor to the possible outcome when only one factor is input at a time (**Figure 6**). The survival probability of *D. shixingense* shows a parabolic trend in bio19, altitude, bio2, and bio9, while it shows a decreasing trend in Soil pH. When bio19 is at 234.43 mm, the survival probability reaches 0.85, indicating the most suitable conditions for bio19; When the altitude is 530.12 m, the survival probability reaches 0.85, indicating the most suitable altitude conditions; When bio2 is at 6.95 °C, the survival probability reaches 0.75, indicating the most suitable daily average temperature conditions; When bio9 is at 11.99 °C, the survival probability reaches 0.82, indicating the most suitable isothermal conditions; The survival probability of *D. shixingense* decreases with the increase of Soil pH. Generally speaking, when the survival probability is greater than 0.5, it indicates that the area is highly suitable for species survival. It can be seen that under current climate conditions, the suitable range for these 5 environmental factors is bio19 between 159.35-319.97mm, Soil pH<7.24, altitude is between 278.64-843.96m, with bio2 at 5.35-8.67 °C and bio9 at 7.08-16.95 °C.

**Figure 7:**
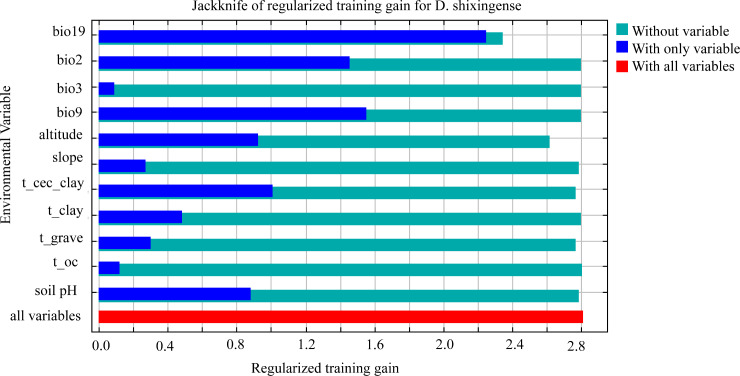
Regularized training gain of the MaxEnt model based on the jackknife test.

Finally, Jackknife tests (**Figure 7**) demonstrated that bio19 had the highest normalized training gain when used in isolation (indicating its unique explanatory power), followed by bio9 and bio2. Conversely, excluding bio19 resulted in the lowest training gain, underscoring its irreplaceable role in explaining distribution patterns. Combined with its dominant contribution rate (32.5%), bio19 emerged as the most critical factor shaping the distribution of *D. shixingense*.

### 3.3 Future distribution of suitable habitats for *D. shixingense*

**Figure 8:**
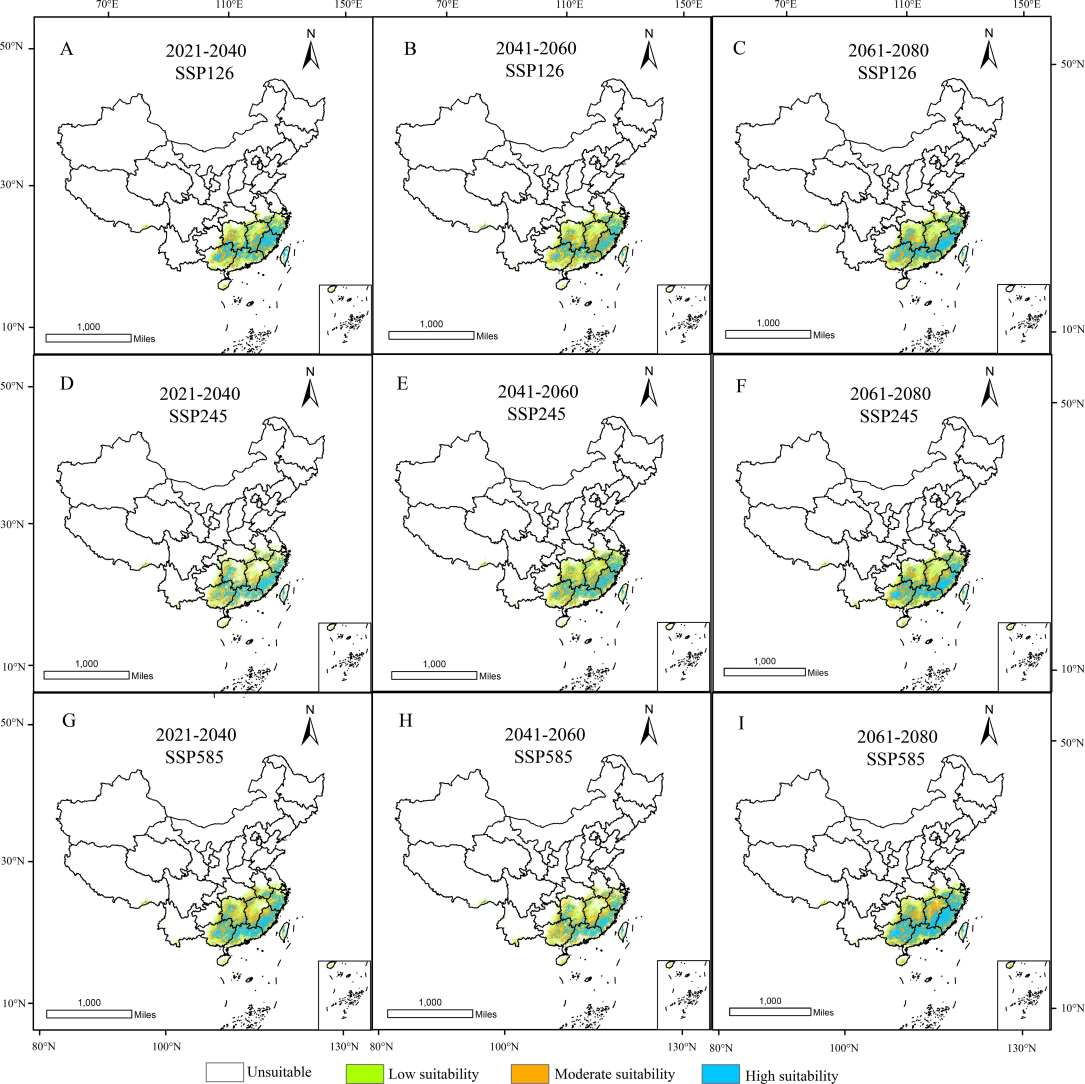
Suitable distribution areas of *D. shixingense* in SSP126 **(A-C)**, SSP245 **(D-F)**, SSP 585 **(G-I)** scenarios in the 2021-2040 **(A, D, G)**, 2041-2060 **(B, E, H)** and 2061-2080 **(C, F, I)**.

In GIS software, visualization was performed to obtain the distribution map of suitable habitats for *D. shixingense* under future climate scenarios (**Figure 8**). Compared to current climatic baselines, the total suitable habitat area exhibits an expansion trend across all future climate scenarios (**Table 4**). For example, during the 2041–2060 period under the SSP126 scenario, the suitable area increased from the current 79.41 × 10^4^ km² to 98.89 × 10^4^ km², with an increase of 19.48 × 10^4^ km², representing a growth rate of 24.53%. Under the SSP245 scenario, the suitable area increased to 93.06 × 10^4^ km², with an increase of 13.65 × 10^4^ km² and a growth rate of 17.19%. Under the SSP585 scenario, the suitable area increased to 92.02 × 10^4^ km², with an increase of 12.61 × 10^4^ km² and a growth rate of 15.88%.

**Table 4:** Changes in the distribution area of *D. shixingense* under different periods and scenarios (10^4^ km²).

Period	Mode	Low suitability	Moderate suitability	High suitability	Overall suitable	contract	contract rate	expansion	Expansion rate
current		38.49	22.65	18.27	79.41				
2021-2040	SSP126	34.72	26.06	29.98	90.76	1.78	2.24	13.1	16.50
	SSP245	33.51	26.57	25.84	85.92	10.03	12.63	16.53	20.82
	SSP585	40.50	32.70	32.45	105.62	2.24	2.82	28.45	35.83
2041-2060	SSP126	44.40	31.50	25.99	98.89	0.52	0.65	20.04	25.24
	SSP245	36.24	30.33	26.49	93.06	1.35	1.7	15.02	18.91
	SSP585	39.32	29.79	22.91	92.02	3.3	4.15	15.95	20.08
2061-2080	SSP126	36.53	28.62	32.88	98.03	0.49	0.62	20.1	25.31
	SSP245	36.38	28.71	30.2	95.29	1.44	1.81	17.13	21.57
	SSP585	30.14	32.09	43.88	106.11	0.49	0.62	27.2	34.25

Under future climate conditions, the total area of low-suitability zones exhibited an overall decreasing trend. The most pronounced change occurred under the SSP585 scenario during 2061-2080, with an area reduction of 8.35 × 10^4^ km². Notably, the area increased by 5.91 × 10^4^ km² and 0.83 × 10^4^ km² under the SSP126 and SSP585 scenarios, respectively, during 2041-2060. The total area of medium-suitability zones showed an increasing trend. The most significant change was observed under the SSP585 scenario during 2021–2040, with an area increase of 10.05 × 10^4^ km². Similarly, the total area of high-suitability zones also exhibited an increasing trend, with a substantial magnitude of growth. The most pronounced change occurred under the SSP585 scenario during 2061-2080, where the area increased by 25.61 × 10^4^ km², representing a 140% growth.

**Figure 9:**
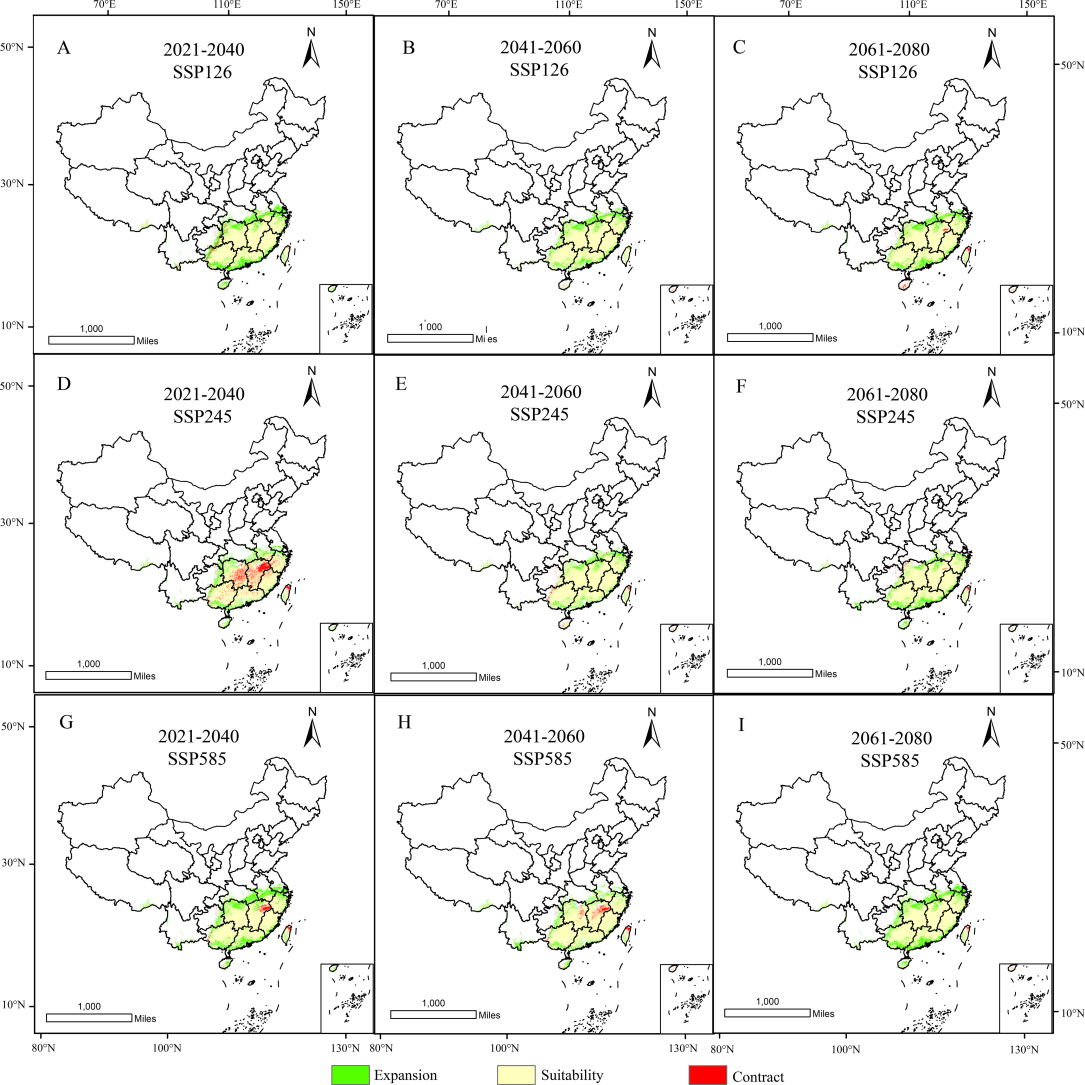
Spatial changes of geographical distribution of *D. shixingense* in SSP126 **(A-C)**, SSP245 **(D-F)**, SSP 585 **(G-I)** scenarios in the 2021-2040 **(A, D, G)**, 2041-2060 **(B, E, H)** and 2061-2080 **(C, F, I)**.

As shown in the table, the contraction rate of suitable habitats for *D. shixingense* under future climate scenarios is relatively small, while the expansion rate is relatively high. During the SSP126 scenario for 2021–2040, the maximum contraction area reached 10.03 × 10^4^ km², with a contraction rate of 12.63%. In contrast, during the SSP126 scenario for 2021–2040, the maximum expansion area reached 28.45 × 10^4^ km², with an expansion rate of 35.83%. As illustrated in **Figure 9**, the expansion regions under future climate scenarios are mainly concentrated in provinces such as Guangdong, Guangxi, Zhejiang, and Anhui, while the contraction regions are primarily located in scattered areas of Taiwan and Guangxi provinces. Overall, the suitable distribution area of *D.shixingense* has not become fragmented due to global warming and has instead undergone a certain degree of expansion.

### 3.4 Centroid shift of *D.shixingense* suitable areas in future periods

**Figure 10:**
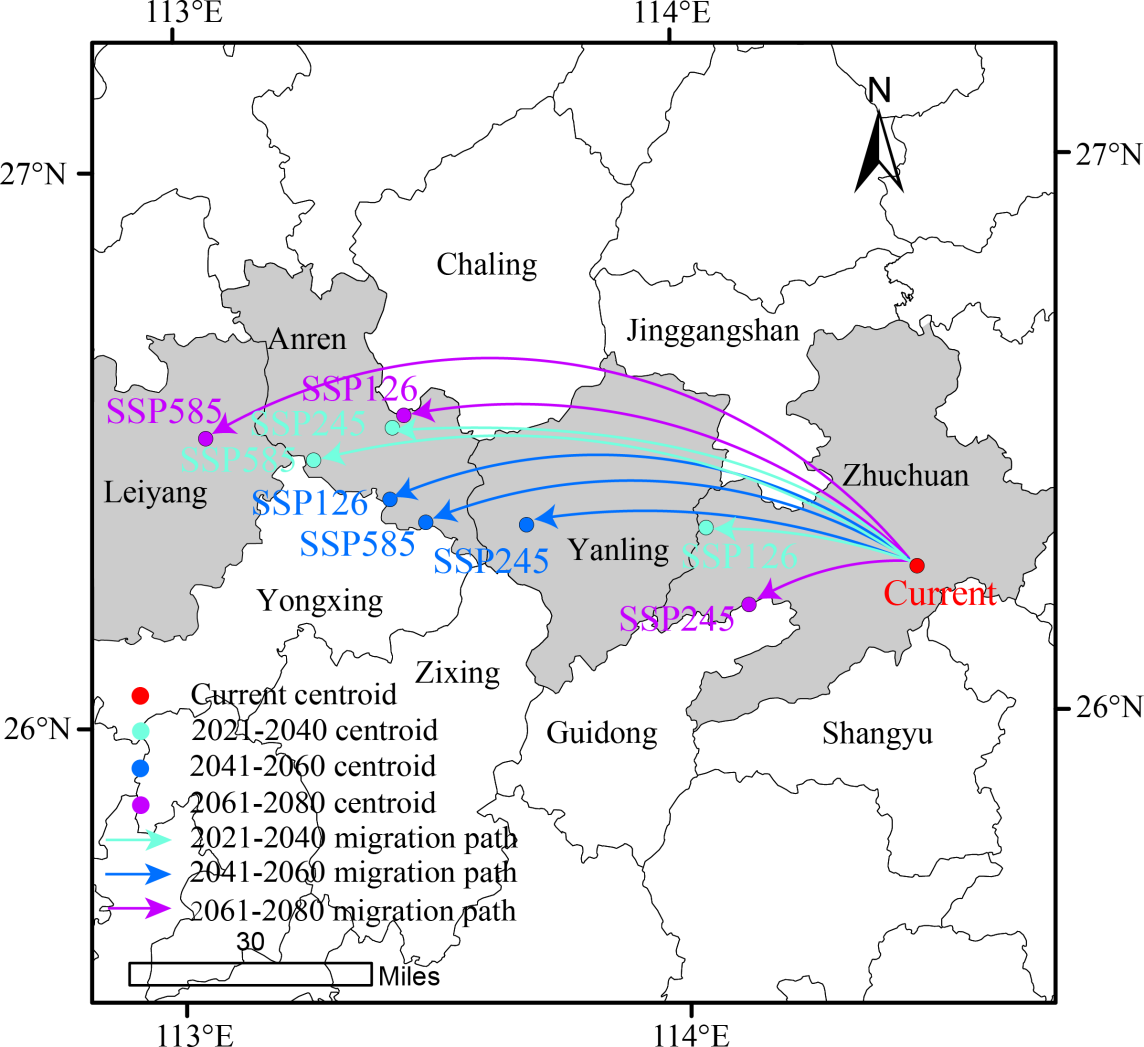
Geographical changes of the central particle in *D. shixingense* under different climatic scenarios and periods.

As shown in **Figure 10**, the centroid shifts of suitable habitats for *D. shixingense* under future climate conditions exhibit spatial variations, but the overall migration direction is westward. Currently, the centroid of the species’ suitable habitat is located in Suichuan County, Ji’an City, Jiangxi Province (114°28’12.92”E, 26°15’50.60”N). Taking the 2061–2080 period under different climate scenarios as an example. In the SSP126 scenario (low emissions). the centroid shifts northwest by 106.66 km to Anren County, Chenzhou City, Hunan Province (113°27’4.70”E, 26°33’21.95”N). In the SSP245 scenario (medium emissions), the centroid shifts northwest by 34.37 km to Guidong County, Chenzhou City, Hunan Province (114°7’59.06”E, 26°12’6.65”N). In the SSP585 scenario (high emissions), the centroid shifts southwest by 144.08 km to Leiyang City, Hengyang City, Hunan Province (113°3’13.88”E, 26°31’11.91”N). These results indicate that under future climate conditions, the combined effects of precipitation and temperature under global warming will drive the centroid of *D.shixingense’s* suitable habitats to shift toward inland regions.

### 3.5 Priority conservation areas for *D.shixingense*

**Figure 11:**
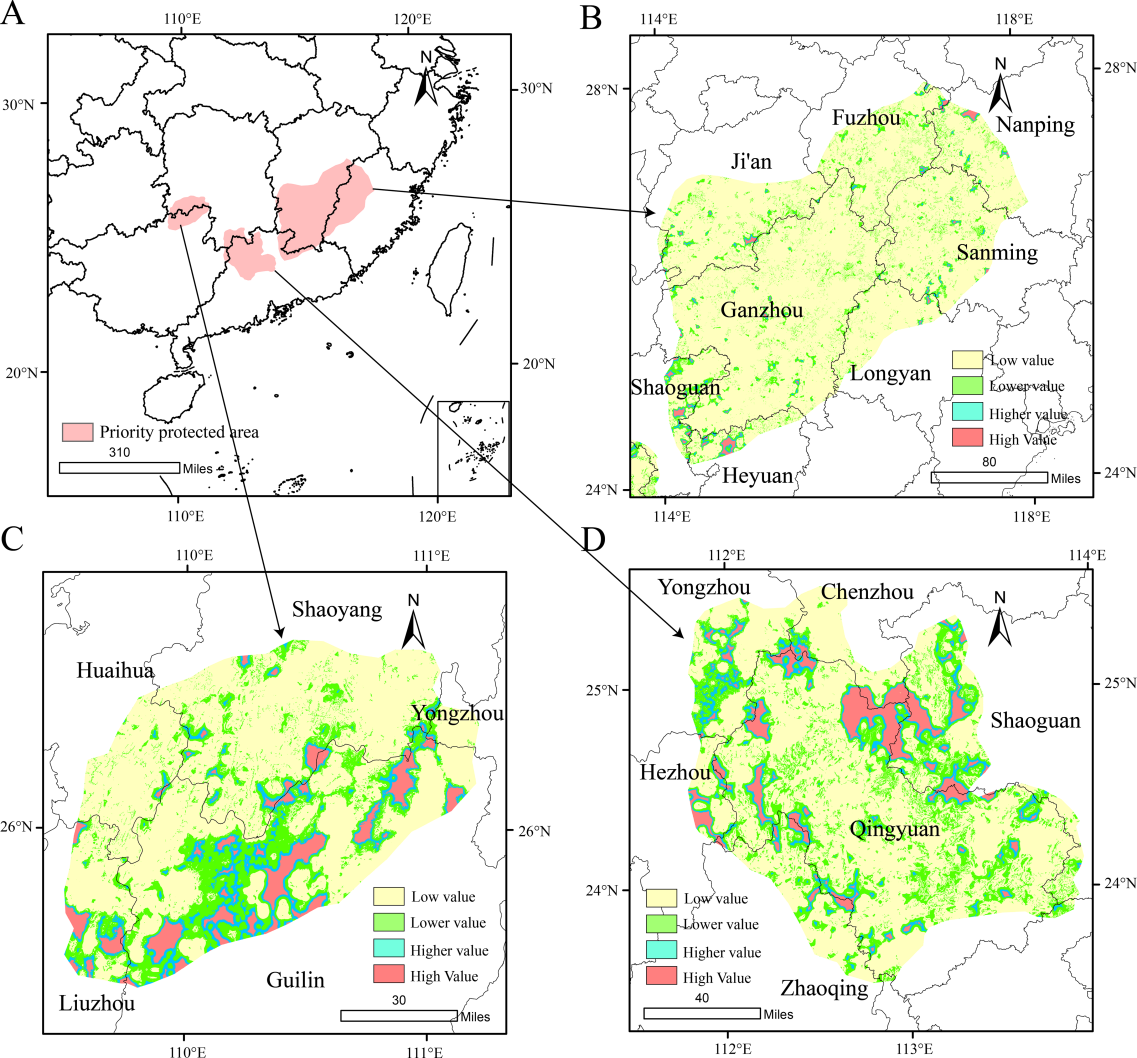
*D. shixingense* Priority Protection Area [**(A)** is the priority protected area for identifying Marxan, **(B-D)** stands for habitat quality in protected areas)].

We employed the Marxan model to preliminarily identify priority conservation areas for *D.shixingense* and conducted spatial smoothing and visualization in a GIS platform (**Figure 11**). The results reveal that priority conservation areas are distributed across five provinces including western Fujian, northern Guangdong, the southeastern edge of Guangxi, the southwestern edge of Jiangxi and southeastern Hunan, with western Fujian and northern Guangdong being the core regions. These areas predominantly overlap with medium-to-high suitability zones under current climatic conditions, further validating the reliability of the model predictions.

Subsequently, the InVEST model was applied to evaluate habitat quality within the Marxan-identified priority areas, and the results were integrated into GIS. Habitat quality was classified into four tiers using the natural breaks method: Low (P < 0.106), Relatively low (0.106 ≤ P < 0.314), Relatively high (0.314 ≤ P < 0.639) and High (P ≥ 0.639). Higher habitat quality tiers indicate greater biodiversity richness and ecological suitability. To optimize conservation efficiency, limited resources should prioritize regions with higher habitat quality. Consequently, this study refined the preliminary conservation areas by excluding low-quality habitats and retaining zones with moderate-to-high quality. The results show that high and relatively high habitat quality areas are primarily distributed in northwestern Guilin City, western Shaoguan City, and northern and northwestern Regions of Qingyuan City. These regions are dominated by stable forest ecosystems with minimal human disturbance, contributing to their superior habitat quality. Notably, although other areas also contain extensive forest-grassland mosaics, their habitat quality is compromised by urbanization and human activities, leading to higher habitat fragmentation. In summary, the final priority conservation areas focus on regions with intact habitat patches and stable ecosystems (e.g., Shixing County and Ruyuan Yao Autonomous County of Shaoguan City, Yangshan County and Lianzhou City of Qingyuan City, Xing’an County of Guilin City, Lianping County of Heyuan City). These areas are prioritized due to their lower conservation costs and enhanced potential for supporting the natural reproduction and growth of *D.shixingense* wild populations.

## 4 Discussion

### 4.1 Evaluation of the Maxent model

The Maxent model, grounded in the principle of maximum entropy (Phillips et al., 2006), predicts species distributions using occurrence data alongside relevant environmental factors. Previous studies have demonstrated that the spatial clustering of occurrence records can significantly influence the model’s predictive accuracy (Yao et al., 2023). To address this, the present study utilized the ENMtools package in R to filter and process the occurrence points. This package eliminates redundant records by randomly removing duplicate occurrences within the same pixel, based on the resolution of the climatic and environmental variables, ensuring that only a single record is retained per pixel and thus minimizing data redundancy. Similar approaches have been adopted by other researchers, who employed the ‘thin’ function in the spThin R package (Aiello-Lammens et al., 2015) and spatial analysis tools in GIS (Yang et al., 2022) to retain only one occurrence per grid cell, yielding favorable results.

The default parameter settings of the Maxent model, established through comprehensive diversity tests on 266 plant and animal species across six major global geographic regions, serve as standard values provided by the developers (Phillips and Dudík, 2008). However, applying these default parameters to other species can sometimes result in overfitting and reduced prediction accuracy (Li et al., 2020). To mitigate this, the ENMeval package in R was used to optimize the Maxent model within this study. Additionally, the ‘kuenm’ package in R enables similar functionality by integrating with MaxEnt, automating the modeling process. This package systematically evaluates various combinations of feature classes (FC) and regularization multipliers (RM) to identify the optimal parameter configuration.

In recent years, bioclimatic, topographic, and soil variables have become standard environmental factors in modeling, but these variables often exhibit high inter-correlation. Such high correlations can introduce redundant information and negatively impact the predictive outcomes (Sun et al., 2021). Accordingly, jackknife testing and Spearman correlation analysis were employed to exclude highly correlated variables, thereby enhancing model accuracy.

Despite Maxent’s many advantages, certain limitations remain. First, as a machine learning-based algorithm, it may occasionally converge on a local optimum rather than a global one, potentially affecting model performance (Elith et al., 2011). Second, the model typically predicts the maximum potential suitable distribution, which may not perfectly coincide with the observed range of the species (Ta et al., 2021). Lastly, while current modeling efforts focus primarily on bioclimatic, topographic, soil, and anthropogenic variables, biotic factors—such as interspecies competition and plant-animal interactions—also play a significant role in shaping species distribution patterns but are often overlooked in related studies (Yang et al., 2025a).

### 4.2 Influence of key environmental variables on the distribution of *D. shixingense*

Understanding the relationship between species’ geographic distributions and environmental factors forms the foundation and a critical step for effective conservation initiatives (Harapan et al., 2022). Climate has long been recognized as a principal determinant of species distribution patterns (Wiens and Zelinka, 2024). As global climate change persists, variables such as temperature, atmospheric CO_2_ concentration, and precipitation exert significant influences on plant growth by affecting key physiological processes like photosynthesis and respiration (Xue et al., 2023). In this study, variables including bio19, bio2, altitude, Soil pH, and bio9 emerged as the most influential in shaping the distribution of *D.shixingense*. The species is most suited to regions characterized by abundant rainfall, warm and humid climates, minimal temperature variation, and relatively low altitudes. Members of the genus Dendrobium have stringent habitat requirements; their distribution is closely linked to climatic conditions, soil type, and vegetation structure. Most species are epiphytic or lithophytic, growing attached to tree branches or within rock crevices (Pan et al., 2022). Because their roots are exposed to the air rather than embedded in soil, their capacity for water uptake is constrained, making them particularly susceptible to water stress (Lü et al., 2023b).

Research has shown that species such as *D.nobile* and *D. officinale* adopt drought avoidance strategies (Lü et al., 2023a). Given that *D. shixingense* belongs to the same genus, it is reasonable to infer that it may employ a similar strategy. The pseudobulb—a specialized structure in these plants—plays a crucial role in storing water, carbohydrates, and minerals, which are essential for survival and growth (Lüttge, 2012). Among the variables considered, bio19 was identified as the most critical, as precipitation during the dry season is vital for replenishing pseudobulb water reserves, thereby supporting subsequent flowering and vegetative growth. Consistent with this, Li (2024) also identified bio19 as the key determinant of ecological suitability for *D. officinale*, suggesting that congeneric species exhibit comparable sensitivities to bioclimatic factors.

Temperature also plays a pivotal role in the growth and development of *Dendrobium* species within Orchidaceae (Hao et al., 2012). These orchids typically inhabit regions with minimal temperature fluctuation and warm, humid climates—a preference likely linked to the pronounced effects of temperature cycles on growth and endogenous hormone-mediated flowering processes (Campos and Kerbauy, 2004; Tang et al., 2020). For *D. shixingense*, optimal bio2 values ranged from 5 to 9°C, while bio9 (isothermality) was optimal between 7 and 17°C.

Topographic factors can indirectly influence the abundance and spatial distribution of plant populations by affecting solar radiation, precipitation, and the redistribution of soil nutrients. *D. shixingense* is primarily found at altitudes between 400 and 600 meters (Chen et al., 2010). According to our model predictions, the most suitable altitude range for this species is 300–850 meters, which further supports the reliability of the model outputs. Moreover, altitude has been identified as a critical factor influencing both the alkaloid content and other secondary metabolites in *D. nobile* (Lu, 2020), highlighting the need for future cultivation practices of *D. shixingense* to pay close attention to the effects of altitude.

The physicochemical properties of soil are known to exert significant influence on both the growth and secondary metabolite production of medicinal plants (Lu et al., 2006). Among these, soil pH is regarded as a key indicator of soil fertility. In this study, when a survival probability threshold of 0.3 was used to define suitable habitat for *Dendrobium shixingense*, the corresponding value for Soil pH was below 6.0. Generally, species in the genus *Dendrobium* thrive best in acidic soils. For example, Meng et al. (2012) achieved successful aseptic cultivation of *D. shixingense* using solid media with a pH of 5.6–5.8, while in their protocorm weight gain experiments, the pH was typically maintained around 4.5 (Meng, 2012). Collectively, these findings underscore the pronounced impact of environmental variables on plant distribution patterns, accentuating the importance of species-specific research to identify which environmental factors are most critical for the survival and reproduction of each species.

The distribution and persistence of species are governed not only by climatic variables, but also by the combined effects of biotic interactions (such as interspecific relationships) and abiotic factors (including anthropogenic activities). These complex ecological processes are often tightly linked to the spatial and temporal heterogeneity of environmental factors (Warren et al., 2001). Although the present study identified climate and topography as the principal determinants of *D. shixingense* distribution, this does not diminish the importance of other variables. This is particularly relevant for epiphytic species, whose establishment and growth are influenced by the characteristics of their host plants (Lin et al., 2017). In our projections, we assumed that soil and topography would remain constant over the next 80 years, which may introduce some degree of bias into the results (Yang et al., 2025a).

### 4.3 Dynamic changes in the distribution of *D. shixingense*

In response to the challenges posed by climate change, plants may persist and grow by adjusting their physiological processes or by shifting their geographic ranges to habitats with more favorable climatic conditions (Kong et al., 2021). As global temperatures rise, the suitable habitats of most species are predicted to shift toward higher latitudes and elecations (Bertrand et al., 2011). *Dendrobium shixingense* is currently restricted in its distribution to Guangdong province and a few adjacent areas bordering Jiangxi (Chen et al., 2010). According to our model projections, the potential suitable range for this species is concentrated in southeastern China—including Guangdong, Fujian, Guangxi, and Jiangxi—primarily within the northern subtropical humid climatic zone, with an estimated area of 79.41 × 10^4^ km². Notably, the model also predicts small patches of suitable habitat along the lower reaches of the Yarlung Tsangpo River.

Previous research has shown that the Yarlung Tsangpo valley harbors a rich and distinctive diversity of orchids (Lin et al., 2013), including numerous *Dendrobium* species such as *D. moniliforme*, *D. chrysotoxum*, and *D. densiflorum* (Yang et al., 2023a). This suggests that the regional climate is highly conducive to the growth of *Dendrobium* species, and we infer that *D. shixingense* could also thrive in this area. Orchidaceae possess highly specialized reproductive traits: their seeds, being minute and lacking endosperm, rely on symbiotic fungi for nutrient acquisition during germination and seedling development (Bidartondo and Read, 2008; Dearnaley et al., 2012). In natural habitats, orchids form mycorrhizal associations with specific fungal taxa, allowing them to obtain the carbohydrates and minerals necessary to complete crucial life stages (Arditti and Ghani, 2000). For instance, *D. huoshanense* exhibits low fruit set due to pollinator limitation, poor seed germination rates in the wild, weak reproductive capability, and slow growth. We therefore hypothesize that similar reproductive constraints may explain the current limited distribution of *D. shixingense* to the Guangdong–Jiangxi border.

Our projections of the future distribution of *D. shixingense* indicate substantial shifts in its potential range under global warming, with the centroid of suitable habitat moving westward. All future scenarios predict an overall increase in suitable area, with the most notable changes occurring in high- and medium-suitability zones, while low-suitability zones show less variation. This pattern underscores the pronounced sensitivity of *D. shixingense* to global temperature changes. Spatially, the species’ suitable range is expected to expand as the climate warms. We speculate that the primary driver of this change is climate warming alleviating *D.shixingense’s* dual constraints of low-temperature stress and drought stress by regulating key bioclimatic factors—precipitation of the coldest quarter (Bio19) and mean diurnal temperature range (Bio2). On one hand, winter warming coupled with altered cold-season precipitation patterns (Bio19) effectively improves hydrothermal conditions in higher-latitude and higher-altitude regions, relieving cold-season drought stress. Scholar study confirms a significant positive correlation between Orchidaceae richness and Bio19 across China (Zhang et al., 2015). On the other hand, appropriate diurnal temperature variation (Bio2) enhances carbon accumulation efficiency by reducing nocturnal respiratory consumption while mitigating water limitations during developmental stages through dew replenishment mechanisms (De & Biswas, 2022). This synergistic hydrothermal optimization process promotes the expansion of suitable habitats toward higher latitudes/altitudes.

Over the past 100 years, the rate of climate warming in China has been slightly higher than the global average (You et al., 2022). Against the backdrop of global warming, extreme high-temperature and cold wave events have become increasingly frequent (Wu et al., 2025). As a crucial component of terrestrial ecosystems, vegetation systems exhibit high sensitivity to such environmental changes (Yang et al., 2025b). Plant growth is often constrained by specific climatic thresholds, and once environmental conditions surpass these tolerance limits, growth rates decline significantly, and in extreme cases, population decline may occur (Hoffman et al., 2018; Luo, 2011). Notably, epiphytic plants are particularly vulnerable to the impacts of climate change compared to other terrestrial plants (Gentry and Dodson, 1987). Studies have shown that at temperatures of 0°C and below, the cells of Dendrobium officinale suffer severe damage, impairing seedling growth and development and even causing death (Wang et al., 2022). While model results suggest that *D. shixingense* may benefit to some extent from climate warming overall, the potential harm caused by frequent extreme weather events, especially to such highly climate-sensitive protected species, warrants significant attention and must not be overlooked.

In this study, the natural breaks method in GIS was used to classify suitability zones, as this method maximizes within-group similarity and between-group differences (Arabameri et al., 2019). However, different classification methods may yield different results. For example, the IPCC’s likelihood classification method (0–0.05–0.33–0.66–1) (Manning, 2006) or using two groups of sample points to define thresholds (sorting each group of sample points in ascending order by output value and selecting points between the top 80% of one group and the bottom 20% of the other group as classification thresholds) (Zhu et al., 2023) are alternative approaches. Therefore, it is necessary to set classification standards appropriate for the species in question and compare these with its actual geographical distribution.

### 4.4 Priority conservation areas for *D. shixingense*

The endangerment mechanisms of rare plants typically include intrinsic factors, such as reproductive barriers and low seed germination rates, as well as extrinsic factors, such as geological natural disasters, pests and diseases, climate change, and human activities leading to habitat degradation and fragmentation (Volis and Deng, 2020). Habitat loss is a primary driver of biodiversity decline, making habitat conservation critically important (Françoso et al., 2015). For species like *D. shixingense*, which have stringent habitat requirements, habitat quality is particularly crucial. The Marxan model is commonly used to identify priority conservation areas. However, when applied at larger scales, it often fails to account for habitat quality and may include areas that are not essential for conservation. In this study, we coupled the Marxan model with the InVEST model to evaluate habitat quality, thereby refining the identification of priority conservation areas.

Our results indicate that priority conservation areas are mainly concentrated in northwestern Guilin City, western Shaoguan City, and northern and northwestern regions of Qingyuan City. These areas overlap significantly with the species’ original habitat, further supporting the validity of the results. Although other regions were not included in the final priority conservation areas, they could still be considered for developing *D. shixingense* cultivation, processing, and related industries. Therefore, our research provides practical insights for the conservation and sustainable development of *D. shixingense*.

## 5 Conclusion

This study utilized an optimized MaxEnt model to predict the suitable habitat of *D. shixingense* under current and future climate conditions. Additionally, the coupling of the Marxan and InVEST models enabled the identification of priority conservation areas. The results showed that the key environmental variables influencing the distribution of *D. shixingense* are bio19, bio2, altitude, Soil pH and bio9. Its primary suitable areas are located in southeastern China, including Fujian, Guangdong, Jiangxi, and Guangxi provinces. Under future climate conditions, the suitable area for *D. shixingense* is expected to expand to some extent, with the distribution centroid shifting inland. Priority conservation areas were predominantly located in Shixing County and Ruyuan Yao Autonomous County of Shaoguan City; Yangshan County and Lianzhou City of Qingyuan City; Xing’an County of Guilin City; and Lianping County of Heyuan City. These findings provide a strong reference for the conservation and sustainable development of *D. shixingense*.

## Data Availability

The original contributions presented in the study are included in the article/**Supplementary Material**. Further inquiries can be directed to the corresponding authors.
