# Supplementary material for: Projecting suitable habitats and prioritizing conservation areas for Dendrobium shixingense under climate change
Source: Front Plant Sci. 2025 Aug 8;16:1620580. doi: 10.3389/fpls.2025.1620580 (PMC12370663; doi:10.3389/fpls.2025.1620580)
Supplement: Supplementary file 1 [file Table1.docx]

Table 1 Factor contribution rate

| Variable | Percent contribution |
| --- | --- |
| bio19 | 32.5 |
| bio2 | 19.9 |
| Altitude | 16.5 |
| Soil pH | 14.6 |
| bio9 | 5.1 |
| t_grave | 4.4 |
| podu | 3.4 |
| t_cec_clay | 2.2 |
| t_clay | 1.1 |
| t_oc | 0.2 |
| bio3 | 0.1 |

Table2 Maxent tuning parameter running results

|  | fc | rm | tune.args | auc.train | cbi.train | auc.diff.avg | auc.diff.sd | auc.val.avg | auc.val.sd | cbi.val.avg | cbi.val.sd | or.10p.avg | or.10p.sd | or.mtp.avg | or.mtp.sd | AICc | delta.AICc | w.AIC | ncoef |
| --- | --- | --- | --- | --- | --- | --- | --- | --- | --- | --- | --- | --- | --- | --- | --- | --- | --- | --- | --- |
| 1 | L | 0.1 | fc.L_rm.0.1 | 0.767189 | 0.819 | 0.217818 | 0.835681 | 0.658835 | 1.208669 | NA | NA | 0.24 | 2.092271 | 0.16 | 1.795996 | 730.1076 | 17.54235 | 8.75E-05 | 10 |
| 2 | LQ | 0.1 | fc.LQ_rm.0.1 | 0.889197 | 0.928 | 0.139197 | 0.804971 | 0.798916 | 0.929411 | NA | NA | 0.16 | 1.795996 | 0.08 | 1.32906 | 770.7759 | 58.2106 | 1.29E-13 | 17 |
| 3 | H | 0.1 | fc.H_rm.0.1 | 0.975904 | 0.95 | 0.136528 | 0.729127 | 0.842731 | 0.740098 | NA | NA | 0.64 | 2.35151 | 0.44 | 2.431789 | NA | NA | NA | 60 |
| 4 | LQH | 0.1 | fc.LQH_rm.0.1 | 0.976024 | 0.929 | 0.135305 | 0.736236 | 0.844177 | 0.747753 | NA | NA | 0.64 | 2.35151 | 0.44 | 2.431789 | NA | NA | NA | 50 |
| 5 | LQHP | 0.1 | fc.LQHP_rm.0.1 | 0.975301 | 0.899 | 0.15323 | 0.758181 | 0.826104 | 0.767337 | NA | NA | 0.68 | 2.285257 | 0.48 | 2.447529 | NA | NA | NA | 54 |
| 6 | LQHPT | 0.1 | fc.LQHPT_rm.0.1 | 0.999639 | 0.897 | 0.162612 | 0.821293 | 0.837068 | 0.821176 | NA | NA | 0.92 | 1.32906 | 0.88 | 1.59198 | NA | NA | NA | 72 |
| 7 | L | 0.5 | fc.L_rm.0.5 | 0.769277 | 0.903 | 0.202515 | 0.825441 | 0.681807 | 1.181721 | NA | NA | 0.24 | 2.092271 | 0.12 | 1.59198 | 722.933 | 10.36771 | 0.003161 | 8 |
| 8 | LQ | 0.5 | fc.LQ_rm.0.5 | 0.847108 | 0.905 | 0.141533 | 0.777326 | 0.765663 | 0.928754 | NA | NA | 0.24 | 2.092271 | 0.04 | 0.96 | 745.1161 | 32.55087 | 4.82E-08 | 14 |
| 9 | H | 0.5 | fc.H_rm.0.5 | 0.934819 | 0.89 | 0.13364 | 0.686143 | 0.821928 | 0.758361 | NA | NA | 0.48 | 2.447529 | 0.16 | 1.795996 | NA | NA | NA | 26 |
| 10 | LQH | 0.5 | fc.LQH_rm.0.5 | 0.93008 | 0.905 | 0.138094 | 0.711227 | 0.817149 | 0.794838 | NA | NA | 0.44 | 2.431789 | 0.16 | 1.795996 | NA | NA | NA | 27 |
| 11 | LQHP | 0.5 | fc.LQHP_rm.0.5 | 0.933253 | 0.857 | 0.141739 | 0.687469 | 0.813052 | 0.768452 | NA | NA | 0.48 | 2.447529 | 0.2 | 1.959592 | NA | NA | NA | 30 |
| 12 | LQHPT | 0.5 | fc.LQHPT_rm.0.5 | 0.941566 | 0.945 | 0.168681 | 0.789267 | 0.795301 | 0.877033 | NA | NA | 0.48 | 2.447529 | 0.28 | 2.199636 | NA | NA | NA | 40 |
| 13 | L | 1 | fc.L_rm.1 | 0.765301 | 0.949 | 0.194111 | 0.794115 | 0.691606 | 1.14785 | NA | NA | 0.24 | 2.092271 | 0.12 | 1.59198 | 723.2278 | 10.66252 | 0.002727 | 7 |
| 14 | LQ | 1 | fc.LQ_rm.1 | 0.837068 | 0.894 | 0.143166 | 0.74696 | 0.76759 | 0.937298 | NA | NA | 0.2 | 1.959592 | 0.08 | 1.32906 | 712.5652 | 0 | 0.563754 | 9 |
| 15 | H | 1 | fc.H_rm.1 | 0.903373 | 0.897 | 0.160017 | 0.734704 | 0.778238 | 0.864122 | NA | NA | 0.375 | 2.32379 | 0.25 | 2.078461 | 849.8938 | 137.3286 | 8.52E-31 | 19 |
| 16 | LQH | 1 | fc.LQH_rm.1 | 0.903293 | 0.882 | 0.145013 | 0.736636 | 0.796426 | 0.857371 | NA | NA | 0.4 | 2.4 | 0.16 | 1.795996 | 902.6282 | 190.063 | 3.02E-42 | 20 |
| 17 | LQHP | 1 | fc.LQHP_rm.1 | 0.905341 | 0.909 | 0.15275 | 0.717103 | 0.78581 | 0.837749 | NA | NA | 0.458333 | 2.391652 | 0.166667 | 1.788854 | 776.7573 | 64.19209 | 6.49E-15 | 17 |
| 18 | LQHPT | 1 | fc.LQHPT_rm.1 | 0.906908 | 0.93 | 0.155803 | 0.758257 | 0.789679 | 0.895274 | NA | NA | 0.36 | 2.35151 | 0.24 | 2.092271 | 843.0307 | 130.4655 | 2.64E-29 | 19 |
| 19 | L | 1.5 | fc.L_rm.1.5 | 0.765502 | 0.929 | 0.189699 | 0.783206 | 0.685462 | 1.116234 | NA | NA | 0.2 | 1.959592 | 0.12 | 1.59198 | 730.2873 | 17.72204 | 7.99E-05 | 7 |
| 20 | LQ | 1.5 | fc.LQ_rm.1.5 | 0.824137 | 0.893 | 0.154332 | 0.744475 | 0.753414 | 0.975244 | NA | NA | 0.16 | 1.795996 | 0.12 | 1.59198 | 715.1435 | 2.578251 | 0.155321 | 8 |
| 21 | H | 1.5 | fc.H_rm.1.5 | 0.880281 | 0.936 | 0.172614 | 0.768037 | 0.749237 | 0.925506 | NA | NA | 0.4 | 2.4 | 0.24 | 2.092271 | 918.7328 | 206.1676 | 9.60E-46 | 20 |
| 22 | LQH | 1.5 | fc.LQH_rm.1.5 | 0.880442 | 0.813 | 0.145894 | 0.751141 | 0.776586 | 0.879128 | NA | NA | 0.32 | 2.285257 | 0.2 | 1.959592 | 843.5344 | 130.9692 | 2.05E-29 | 19 |
| 23 | LQHP | 1.5 | fc.LQHP_rm.1.5 | 0.887189 | 0.92 | 0.156084 | 0.739095 | 0.76414 | 0.854506 | NA | NA | 0.416667 | 2.366432 | 0.208333 | 1.949359 | 849.8135 | 137.2482 | 8.87E-31 | 19 |
| 24 | LQHPT | 1.5 | fc.LQHPT_rm.1.5 | 0.890562 | 0.954 | 0.158136 | 0.75632 | 0.768742 | 0.881563 | NA | NA | 0.375 | 2.32379 | 0.25 | 2.078461 | 908.9619 | 196.3967 | 1.27E-43 | 20 |
| 25 | L | 2 | fc.L_rm.2 | 0.76253 | 0.842 | 0.182252 | 0.778147 | 0.681406 | 1.082334 | NA | NA | 0.24 | 2.092271 | 0.08 | 1.32906 | 740.6273 | 28.06208 | 4.54E-07 | 7 |
| 26 | LQ | 2 | fc.LQ_rm.2 | 0.810482 | 0.927 | 0.168047 | 0.770917 | 0.73241 | 1.036345 | NA | NA | 0.2 | 1.959592 | 0.12 | 1.59198 | 723.0507 | 10.48548 | 0.00298 | 8 |
| 27 | H | 2 | fc.H_rm.2 | 0.850884 | 0.932 | 0.172562 | 0.758182 | 0.728353 | 0.940688 | NA | NA | 0.28 | 2.199636 | 0.16 | 1.795996 | 784.5732 | 72.00795 | 1.30E-16 | 16 |
| 28 | LQH | 2 | fc.LQH_rm.2 | 0.861767 | 0.934 | 0.146941 | 0.720761 | 0.761928 | 0.866835 | NA | NA | 0.28 | 2.199636 | 0.12 | 1.59198 | 720.9895 | 8.424274 | 0.008352 | 13 |
| 29 | LQHP | 2 | fc.LQHP_rm.2 | 0.863655 | 0.958 | 0.166059 | 0.728131 | 0.729639 | 0.851331 | NA | NA | 0.28 | 2.199636 | 0.16 | 1.795996 | 791.8854 | 79.32012 | 3.36E-18 | 17 |
| 30 | LQHPT | 2 | fc.LQHPT_rm.2 | 0.866104 | 0.953 | 0.17041 | 0.744083 | 0.726345 | 0.864501 | NA | NA | 0.32 | 2.285257 | 0.16 | 1.795996 | 820.3784 | 107.8131 | 2.19E-24 | 18 |
| 31 | L | 2.5 | fc.L_rm.2.5 | 0.757149 | 0.751 | 0.17546 | 0.76084 | 0.685221 | 1.060771 | NA | NA | 0.24 | 2.092271 | 0.08 | 1.32906 | 750.2906 | 37.72533 | 3.62E-09 | 6 |
| 32 | LQ | 2.5 | fc.LQ_rm.2.5 | 0.794056 | 0.936 | 0.176899 | 0.774009 | 0.716506 | 1.064432 | NA | NA | 0.24 | 2.092271 | 0.12 | 1.59198 | 727.0136 | 14.44837 | 0.000411 | 7 |
| 33 | H | 2.5 | fc.H_rm.2.5 | 0.822831 | 0.897 | 0.170883 | 0.743797 | 0.715422 | 0.964603 | NA | NA | 0.24 | 2.092271 | 0.04 | 0.96 | 745.9619 | 33.39665 | 3.16E-08 | 10 |
| 34 | LQH | 2.5 | fc.LQH_rm.2.5 | 0.851767 | 0.947 | 0.15216 | 0.727421 | 0.750562 | 0.88942 | NA | NA | 0.28 | 2.199636 | 0.08 | 1.32906 | 714.3187 | 1.753497 | 0.234597 | 11 |
| 35 | LQHP | 2.5 | fc.LQHP_rm.2.5 | 0.84502 | 0.944 | 0.159692 | 0.712867 | 0.725783 | 0.856775 | NA | NA | 0.28 | 2.199636 | 0.04 | 0.96 | 721.1322 | 8.566908 | 0.007777 | 10 |
| 36 | LQHPT | 2.5 | fc.LQHPT_rm.2.5 | 0.84502 | 0.944 | 0.159692 | 0.712867 | 0.725783 | 0.856775 | NA | NA | 0.28 | 2.199636 | 0.04 | 0.96 | 721.1322 | 8.566908 | 0.007777 | 10 |
| 37 | L | 3 | fc.L_rm.3 | 0.755984 | 0.751 | 0.177574 | 0.757765 | 0.681044 | 1.0598 | NA | NA | 0.24 | 2.092271 | 0.08 | 1.32906 | 755.737 | 43.17173 | 2.38E-10 | 5 |
| 38 | LQ | 3 | fc.LQ_rm.3 | 0.787671 | 0.954 | 0.174629 | 0.764491 | 0.7151 | 1.056183 | NA | NA | 0.24 | 2.092271 | 0.08 | 1.32906 | 729.1112 | 16.54592 | 0.000144 | 6 |
| 39 | H | 3 | fc.H_rm.3 | 0.796165 | 0.93 | 0.174547 | 0.74051 | 0.682309 | 0.965029 | NA | NA | 0.28 | 2.199636 | 0.04 | 0.96 | 771.7539 | 59.18863 | 7.91E-14 | 9 |
| 40 | LQH | 3 | fc.LQH_rm.3 | 0.843534 | 0.939 | 0.152607 | 0.736286 | 0.741004 | 0.899672 | NA | NA | 0.28 | 2.199636 | 0.08 | 1.32906 | 720.1945 | 7.629224 | 0.012429 | 10 |
| 41 | LQHP | 3 | fc.LQHP_rm.3 | 0.836867 | 0.946 | 0.165328 | 0.741856 | 0.711727 | 0.895727 | NA | NA | 0.36 | 2.35151 | 0.04 | 0.96 | 728.9797 | 16.41449 | 0.000154 | 8 |
| 42 | LQHPT | 3 | fc.LQHPT_rm.3 | 0.836867 | 0.946 | 0.165328 | 0.741856 | 0.711727 | 0.895727 | NA | NA | 0.36 | 2.35151 | 0.04 | 0.96 | 728.9797 | 16.41449 | 0.000154 | 8 |
| 43 | L | 3.5 | fc.L_rm.3.5 | 0.756787 | 0.839 | 0.176002 | 0.809465 | 0.661285 | 1.058602 | NA | NA | 0.28 | 2.199636 | 0.12 | 1.59198 | 763.4751 | 50.90987 | 4.97E-12 | 5 |
| 44 | LQ | 3.5 | fc.LQ_rm.3.5 | 0.786185 | 0.941 | 0.174767 | 0.772546 | 0.706426 | 1.057826 | NA | NA | 0.24 | 2.092271 | 0.08 | 1.32906 | 734.6399 | 22.07461 | 9.07E-06 | 6 |
| 45 | H | 3.5 | fc.H_rm.3.5 | 0.763956 | 0.935 | 0.174109 | 0.722774 | 0.658876 | 0.956343 | NA | NA | 0.24 | 2.092271 | 0.04 | 0.96 | 790.5947 | 78.02946 | 6.42E-18 | 7 |
| 46 | LQH | 3.5 | fc.LQH_rm.3.5 | 0.833253 | 0.898 | 0.157801 | 0.730874 | 0.726627 | 0.913671 | NA | NA | 0.32 | 2.285257 | 0.04 | 0.96 | 730.2155 | 17.65026 | 8.29E-05 | 9 |
| 47 | LQHP | 3.5 | fc.LQHP_rm.3.5 | 0.827229 | 0.917 | 0.17446 | 0.771648 | 0.689317 | 0.928218 | NA | NA | 0.32 | 2.285257 | 0.04 | 0.96 | 752.8518 | 40.2865 | 1.01E-09 | 8 |
| 48 | LQHPT | 3.5 | fc.LQHPT_rm.3.5 | 0.827229 | 0.917 | 0.17446 | 0.771648 | 0.689317 | 0.928218 | NA | NA | 0.32 | 2.285257 | 0.04 | 0.96 | 752.8518 | 40.2865 | 1.01E-09 | 8 |
| 49 | L | 4 | fc.L_rm.4 | 0.7551 | 0.721 | 0.185266 | 0.834961 | 0.626667 | 1.055355 | NA | NA | 0.28 | 2.199636 | 0.16 | 1.795996 | 771.7509 | 59.18561 | 7.93E-14 | 5 |
| 50 | LQ | 4 | fc.LQ_rm.4 | 0.784618 | 0.817 | 0.180875 | 0.765561 | 0.689157 | 1.052985 | NA | NA | 0.24 | 2.092271 | 0.12 | 1.59198 | 741.4652 | 28.89995 | 2.99E-07 | 6 |
| 51 | H | 4 | fc.H_rm.4 | 0.753494 | 0.887 | 0.177387 | 0.718193 | 0.643835 | 0.959951 | NA | NA | 0.2 | 1.959592 | 0.04 | 0.96 | 800.2697 | 87.70446 | 5.09E-20 | 6 |
| 52 | LQH | 4 | fc.LQH_rm.4 | 0.827831 | 0.85 | 0.165778 | 0.737027 | 0.7151 | 0.939089 | NA | NA | 0.4 | 2.4 | 0.08 | 1.32906 | 737.1543 | 24.58904 | 2.58E-06 | 8 |
| 53 | LQHP | 4 | fc.LQHP_rm.4 | 0.808594 | 0.791 | 0.184236 | 0.795851 | 0.658072 | 0.93834 | NA | NA | 0.24 | 2.092271 | 0.04 | 0.96 | 776.1109 | 63.54562 | 8.96E-15 | 7 |
| 54 | LQHPT | 4 | fc.LQHPT_rm.4 | 0.808594 | 0.791 | 0.184236 | 0.795851 | 0.658072 | 0.93834 | NA | NA | 0.24 | 2.092271 | 0.04 | 0.96 | 776.1109 | 63.54562 | 8.96E-15 | 7 |
